# Reciprocal transplantation experiments reveal local adaptation of seaweed-associated bacteria

**DOI:** 10.1093/ismeco/ycaf205

**Published:** 2025-11-10

**Authors:** Shauna Corr, Chris Lowe, Michiel Vos

**Affiliations:** European Centre for Environment and Human Health, University of Exeter Medical School, Environment and Sustainability Institute, Penryn, Cornwall, TR10 9FE, United Kingdom; Plymouth Marine Laboratory, Marine Ecology and Society, Plymouth, Devon, PL1 3DH, United Kingdom; The Cornish Seaweed Company Ltd, Rosuick Farm, St Martin, Helston, Cornwall, TR12 6DZ, United Kingdom; European Centre for Environment and Human Health, University of Exeter Medical School, Environment and Sustainability Institute, Penryn, Cornwall, TR10 9FE, United Kingdom

**Keywords:** microbiome, local adaptation, reciprocal transplant, *Palmaria palmata*, *Fucus serratus*, macroalgae

## Abstract

Seaweed microbiomes are diverse and frequently species-specific. By actively attracting and repelling settling bacteria through exuded metabolites, seaweeds are thought to exert a strong selective pressure on their microbiomes. However, to what degree seaweed-associated bacteria are adapted to their host has received little attention. Here, we retrieve cultivable seaweed bacterial communities from *Palmaria palmata* (Dulse) and *Fucus serratus* (Serrated Wrack) and use reciprocal transplant experiments to test whether bacterial isolates have the greatest fitness on their host seaweed species. We used agar derived from host seaweed extracts for bacterial isolation, which was found to be superior to a generic marine agar formulation based on both 16S rRNA gene amplicon alpha- and beta-diversity comparisons to uncultured samples. We then demonstrate that bacterial isolates from both seaweed species exhibit higher fitness in media derived from their native host compared to a non-native host. Although epibacterial fitness varied between hosts, bacterial isolates on average outperformed non-native counterparts in their native environment. By integrating amplicon sequencing with laboratory experiments, we demonstrate that bacteria are locally adapted to their seaweed host species. These findings contribute to the growing body of research exploring the evolutionary and ecological drivers that shape bacterial communities, with implications for ecosystem management, disease control, and microbial biotechnology.

## Introduction

All multicellular organisms harbour distinct microbial assemblages which are increasingly recognised as being integral to host health and development [1–3]. Divergent host species tend to have dissimilar microbiomes [4], with hosts and microbiota exhibiting parallel evolutionary histories in a range of systems [5, 6]. Whilst host genetics is known to influence microbiomes [7], the adaptation of bacteria to their hosts has received relatively little attention. This is problematic, as understanding how divergent selection imposed by local conditions, specifically host identity, structures microbial populations and communities is a major goal in microbial ecology [8–13].

Much of our knowledge of microbiome-host associations has been based on sequencing surveys, which are mainly correlative in nature and cannot always elucidate the underlying ecological and evolutionary processes driving observed patterns. Instead, experiments under controlled conditions are more suited to explicitly test whether hosts exert selective pressures on their associated bacteria, potentially leading to host-specific adaptation. A main experimental framework to study bacterial host adaptation is that of local adaptation [10, 14]. Here, reciprocal transplant experiments are used to test whether isolates demonstrate higher fitness in their native compared to non-native environments and whether native isolates outperform non-native isolates within this context [10, 14]. This experimental approach has revealed evidence for local adaptation in a range of systems [9, 11, 15–17], but to our knowledge has not yet been applied in a marine microbiology context.

Here, we test whether epibacteria are adapted to their seaweed hosts. Seaweeds (macroalgae) encompass a wide array of life histories, morphologies, and chemical profiles, harbouring highly diverse species-specific microbiomes thought to be key to host functioning [1]. Their surfaces are typically covered with a complex mix of polysaccharides, proteins, and secondary metabolites, which act as both nutrient sources and chemical defence [18–21], [22]. This chemical landscape varies among seaweed taxa and may selectively promote the growth of certain bacteria whilst inhibiting others [18–22]. For instance, exuded alginates may serve as a carbon source for bacteria capable of degrading them, effectively filtering for taxa with these metabolic traits [22]. Other seaweed compounds may exhibit antimicrobial properties, thereby excluding nontolerant species [22]. Therefore, the pronounced differences in bacterial community composition between co-located seaweed hosts suggest that host-mediated selection may be a dominant factor shaping these communities.

To test whether bacteria are adapted to their seaweed host species, we performed a multisite reciprocal transplant experiment [10, 14] using two common European seaweed species known to harbour distinct microbiomes [23, 24]: *Palmaria palmata* (Dulse; Class Rhodophyta) and *Fucus serratus* (Serrated Wrack; Class Phaeophycea; Fig. S1). First, we isolated bacteria from the host using seaweed-derived agar, which we demonstrate maximises the isolation efficiency of seaweed bacterial communities. Second, we reveal through reciprocal transplants that bacterial isolates exhibit greater fitness in native compared to non-native host environments, as well as outperforming their non-native counterparts, a signature consistent with local adaptation.

## Materials and methods

### Culturing bacterial isolates for a reciprocal two-species transplant experiment

**Figure 1:**
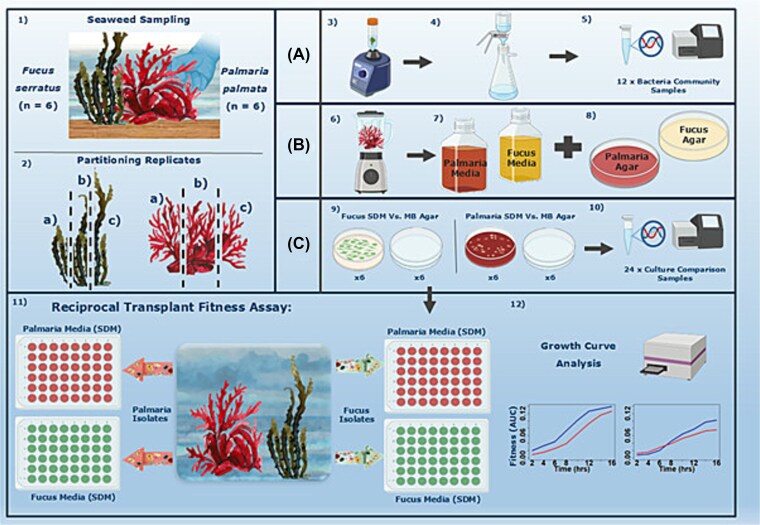
Experimental design of the reciprocal two-species transplant experiment. The seaweeds, *P. Palmata* and *F. Serratus* were sampled from the same location (*n =* 6 for each species) (1) with each seaweed replicate sub-sectioned into three parts: A, B, and C. Section a was used to sample the bacterial community of each seaweed replicate via sonication (3) and filtering (4), these samples were then sent for 16S rRNA gene amplicon sequencing (5). Section B was used to make seaweed-derived media (SDM), seaweeds were blended with sea water (6) and autoclaved to make liquid broth for subsequent reciprocal transplant fitness assays (7) or were set with agar (8) to culture bacterial communities for later use. Section C was used to compare culturing techniques. Seaweeds were imprinted either on SDM or generic MB agar (9) and the resultant communities sent for 16S rRNA gene amplicon sequencing (10). 24 bacterial isolates were taken from each SDM plate for use in reciprocal transplant fitness assays (24 × 6 = 144 for each seaweed host), whereby each isolate was grown in either its native host’s SDM or non-native SDM (11). Growth of each isolate was measured over 24 h and used to compare the fitness of isolates between a native (blue) and non-native (red) environment (12). Created with BioRender.com.

*P. palmata* (*n* = 6) and *F. serratus* (*n =* 6) were sampled from Plymouth Sound, UK (50° 21′ 50.4216",—4° 8' 44.0448") in December 2022. Individuals were collected in zip-lock bags and transported to Plymouth Marine Laboratory for immediate processing. Thalli were rinsed with sterile filtered seawater (SFSW) to remove loosely attached bacteria and macro-epibionts. Each individual *P. palmata* and *F. serratus* seaweed replicate was partitioned into two halves (Fig. 1). The first half of each seaweed individual was used to make Seaweed-Derived Medium (in agar and liquid form). The agar consisted of 10 ± 0.05 g of seaweed material homogenised in 100 ml of SFSW with 3 ± 0.05 g of agar (Oxoid, United Kingdom), whilst the liquid media consisted of 5 ± 0.05 g of seaweed material homogenised in 100 ml of SFSW. Both homogenates were autoclaved for sterility.

The second half of each seaweed replicate was reserved to obtain bacterial isolates and extract bacterial DNA for 16S rRNA gene amplicon sequencing (see below). Each of the six seaweed replicates from each of the two host species were plated on the Seaweed-Derived Agar from each corresponding seaweed replicate, as well as on Difco Marine Broth agar (Oxoid, United Kingdom). The tip of each seaweed frond was imprinted on each agar plate for 1 min (for surface areas, see Table S1). Plates were incubated at 11°C (sea surface temperature at the time of sampling) for seven days. To obtain the isolates for reciprocal transplantation, a line was drawn across each of the Seaweed-Derived Agar plates, and the first 24 colonies within 0.5 cm of that line were picked and grown overnight in Difco Marine Broth (*P. palmata: n =* 6 × 24 = 144; *F. serratus: n =* 6 × 24 = 144*)*. The remaining colonies from each Seaweed-Derived and Marine Broth plates were swabbed for a pooled, plate-level analysis of epibacterial diversity using 16S rRNA gene amplicon sequencing. Colonies were cryopreserved at −80°C prior to DNA extraction (see below).

### Reciprocal transplant experiment

To assess the extent to which bacterial isolates were adapted to their hosts, the growth rates of each isolate grown in native and non-native Seaweed-Derived Medium were compared. 180 μL of *F. serratus* or *P. palmata* liquid Seaweed-Derived Medium was pipetted into sterile 96-well plates and inoculated in triplicate with 20 μL of mature cultures (obtained via overnight incubation at 25°C on an orbital shaker) of the *F. serratus* and *P. palmata* bacterial isolates. There were 144 bacterial isolates for each seaweed species, and each isolate was grown in triplicate in both media types, resulting in a total of 1728 cultures. Cultures were incubated at 11°C on an orbital shaker to maintain homogeneity for OD readings. A microplate reader (BMG LABTECH, Germany) was used to measure absorbance at 600 nm every 2 h over a 24-h period. Growth curves were constructed using the package '*growthcurver*' [25] in R v4.3.2 [26] through R Studio interface v2023.3.1.446 [27]. Area Under the Curve (AUC) was used as a metric of fitness as it combines information on the growth curve’s lag phase, growth rate, and carrying capacity [28], with model fit evaluated through sigma values. The response of fitness to each Seaweed-Derived Medium for each bacterial isolate was determined as delta AUC (i.e. the difference in growth between *F. serratus* and *P. palmata* Seaweed-Derived Media). Delta AUC was determined as the slope of a linear model fitted for each isolate using the 'lmList' function from the 'nlme' package [29]. To assess if the origin of the isolates significantly affected their growth response within these environments, linear mixed effect models were performed using the '*lmer*' function from the '*lme4*' package [30]. Growth response (delta AUC) was fitted as the response variable, with isolate origin fitted as a fixed factor. Seaweed host replicate was included as a random effect to account for host-level variation not explained by isolate origin. All isolates exhibiting zero growth were excluded from the analysis. Post hoc Tukey tests using the ‘*emmeans*’ package [31] were conducted to compare differences between origins, with the false discovery rate method (*fdr*) used to adjust *P* values for multiple testing [32]. Assumptions of normality and homoscedasticity were validated by visual inspection of the models’ residual plots.

### 16S rRNA-based microbiome characterisation

Bacterial communities were extracted by placing 3 ± 0.05 g of each seaweed, 45 ml of SFSW and sterile glass beads in a Falcon tube, which was then sonicated and vortexed for 3 min each to dislodge bacteria [33]. To remove any microalgae, samples were filtered through sterile 5 μm filters, and cells in the subsequent filtrate were collected on 0.2 μm nitrocellulose filters and stored at −80°C prior to DNA extraction. DNA from Seaweed-Derived agar plates, Marine Broth agar plates and the suspended communities taken directly from each seaweed were extracted using a Qiagen DNeasy PowerWater Kit and Zymo Clean & Concentrator-25 Kit following standard protocols. DNA concentrations were quantified using the Invitrogen Qubit DNA High Sensitivity Kit and DNA integrity assessed by visual inspection on 1% agarose gels. Sequencing of the 16S rRNA gene V4 region using the 515F (GTGYCAGCMGCCGCGGTAA)- 806R (GGACTACNVGGGTWTCTAAT) primer set [34] was completed by the University of Exeter Sequencing Service using an Illumina MiSeq platform.

Amplicons were processed using QIIME2 [35]. The '*Cutadapt*' tool v4.0 was used to trim raw fastq files for the presence of Illumina sequences [36] and the DADA2 pipeline v2023.9.0 to denoise sequences to create ASVs [37]. The first 10 bp of the forward and 50 bp of the reverse reads were trimmed, followed by dereplication, merging of the paired reads and removal of chimeric sequences. ASV taxonomy was assigned via the q2-feature classifier using the Silva-ARB v138.1 database [38, 39], with the SILVA database evaluated using the '*RESCRIPt*' plug-in [40]. '*FastTree*' was used to estimate a phylogenetic tree [41] to calculate UniFrac distances between communities [42]. Differential abundance analyses were completed via ANCOM-BC using the '*q2-composition*' plugin [43, 44] to identify ASVs driving differences between uncultured epibacterial samples.

Samples were rarefied (Fig. S2) and Shannon diversity [45] was calculated using the R packages '*qiime2R*' [46], '*phyloseq*' [47], and '*vegan*' [48] in R v4.3.2 [26] and plotted using the '*ggplot2*' package [49]. Diversity metrics were compared using Kruskal–Wallis and Wilcoxon rank sum tests, whilst comparisons with native environments were compared via ANOVA and Dunnett's tests. Weighted UniFrac distances and Principal Coordinate Analysis plots were used to visualise beta diversity [42]. To test for differences in bacterial community composition between treatments and species, we performed PERMANOVAS using '*vegan::adonis2*' with 9999 permutations and conducted pairwise PERMANOVA comparisons between all species-treatment combinations using 'pairwaise.adonis2' with 9999 permutations. *P* values were adjusted using the *fdr* method [32]. Homogeneity of group dispersions was tested using 'vegan::betadisper'.

## Results

### Seaweed epibacteria are most efficiently retrieved using host-derived medium

Effective retrieval of isolates is essential prior to performing experiments on host-associated bacteria. Since a growth medium that closely mirrors the host is expected to result in a more realistic representation of seaweed-adapted bacteria, we created growth media containing homogenates of either of the two host seaweed species (*P. palmata* and *F. serratus*) and compared 16S rRNA gene diversity of isolates grown on this Seaweed-Derived Medium with standard Marine Broth Agar as well as with uncultured bacterial diversity.

**Figure 2:**
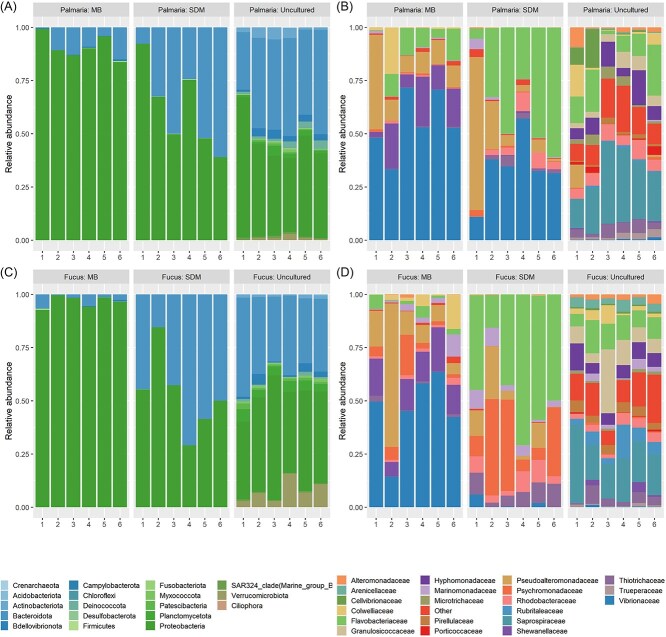
Relative abundance (%) of bacterial taxa at phylum (*P. Palmata* (A), *F. Serratus* (C)) and family (*P. Palmata* (B), *F. Serratus* (D)) level for uncultured bacterial community samples, bacterial community samples obtained via seaweed-derived medium (SDM), and via marine broth agar (MB) for both *F. Serratus* and *P. Palmata*. A total of 3 464 053 V4 16S rRNA gene sequence reads (average: 126 849 (64 124–132 114)) were generated with 1098 ASVs assigned across all samples (18 phyla, 33 classes, 70 orders, 107 families, 198 genera). Each bar depicts a sample, with taxonomic groups colour-coded (see legend). Abundances are normalised as the percentage of total microbial sequences within each sample; only taxa comprising >2.5% of the total abundance appearing in 5% of samples are shown individually, while rare taxa are combined as 'other.'

In all treatments, 16S rRNA gene analysis found *Proteobacteria (Pseudomonadota)* and *Bacteroidota* to be the most dominant phyla, with relative abundances more similar between uncultured and Seaweed-Derived Medium than between Marine Broth Agar treatments (Fig. 2A and C). *Saprospiraceae, Granulosicoccaceae*, and *Flavobacteriaceae* were the most prevalent families for the *F. serratus* uncultured treatment (Fig. 2D), with *Saprospiraceae, Flavobacteriaceae*, and *Hyphomonadaceae* highest for the *P. palmata* uncultured treatment (Fig. 2B; Fig. S3). At the family level, results from the culture-dependent treatments were more variable, with *Flavobacteriaceae, Psychromonadaceae*, and *Pseudoalteromonadaceae* in high abundance for the *F. serratus* Seaweed-Derived Medium treatment and *Flavobacteriaceae, Vibrionaceae*, and *Pseudoalteromonadaceae* highest in the *P. palmata* Seaweed-Derived Medium treatment (Fig. 2B and D). Marine Broth Agar treatments were dominated by *Vibrionaceae, Pseudoalteromonadaceae*, and *Shewanellaceae* for both seaweed species (Fig. 2B and D).

**Figure 3:**
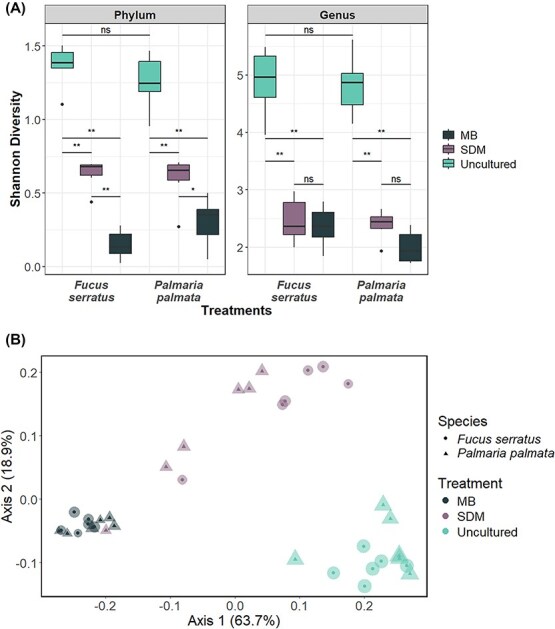
Comparison of alpha and beta diversity of ASVs obtained via 16S rRNA gene amplicon sequencing between uncultured (blue) and cultured bacterial communities via seaweed-derived medium (SDM: Purple) and marine broth agar (MB: Black) of *F. Serratus* and *P. Palmata.* (A): Boxplots displaying variation in Shannon diversity at both the genus and phylum level. Significance levels between treatments are indicated by asterisks: Not significant (ns), *P <* .05 (*)*, P <* .01 (**), *P <* .001 (***) and *P <* .0001 (****) (B): Principal coordinate analysis plot based on weighted UniFrac distances with point size corresponding to Shannon diversity indicating that SDM samples are more phylogenetically similar to uncultured samples than MB samples.

Seaweed-Derived Medium consistently outperformed Marine Broth Agar in capturing a broader and more representative subset of the native bacterial communities (Fig. 3). While both culture-based methods retrieved only a modest fraction of total diversity (Fig. 2), bacterial communities grown on Seaweed-Derived Medium yielded a significantly greater Shannon diversity than Marine Broth Agar, and so more closely resembled the uncultured bacterial assemblages in terms of alpha diversity at higher taxonomic levels such as Phylum (Kruskal–Wallis: H(5) = 30.50, *P* < .0001; Pairwise Wilcox: *P <* .05). Alpha diversity between the two cultured treatments were indistinguishable at the genus level, due to a lower level of taxonomic assignment (Table S2, Fig. 3A). Beta diversity significantly differed between the bacterial communities derived from the two host species [23, 24] (Table S2, Fig. S3; PERMANOVA: F = 3.84, R^2^ = 0.277, *P* = .0039) and for communities derived using the two culture-based methods (Seaweed Derived Medium & Marine Broth Agar; PERMANOVA: F_5_ = 22.33, R^2^ = 0.79, *P <* .001). Importantly, based on UniFrac distances, the taxonomic composition of the bacterial communities isolated using Seaweed-Derived Medium was more similar to those derived from non-uncultured samples than those derived using generic Marine Broth Agar for both seaweed species (Fig. 3B; Fig. S4). The patterns in species composition/beta-diversity were visualised using PCoA. PCoA axes 1 (64%) and 2 (19%) explained 83% of the total variation in the beta-diversity similarity scores. Samples derived from Marine Broth Agar and uncultured samples (Fig. 3B; Fig. S5) formed distinct clusters and were separated by the greatest distance along PCoA 1; samples derived via Seaweed-Derived Media were intermediate between these two groups but did not form a discrete cluster. These patterns underscore the limitations of generic media in recovering realistic representations of host-specific bacterial diversity.

### Reciprocal growth rate assay reveals local adaptation of seaweed epibacteria

**Figure 4:**
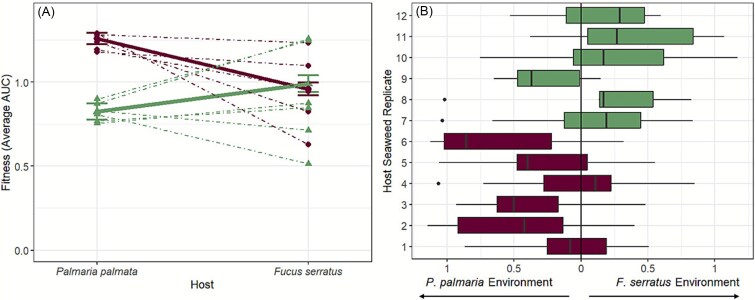
(A) Average fitness (AUC score) of bacterial isolates from each seaweed host replicate in native and non-native *F. Serratus (*dashed green) and *P. Palmata* (dashed red) environments, with total average fitness of all combined bacterial isolates overlaid (solid lines), indicating a significant interaction between host environment and epibacterial isolate origin. (B) Average fitness of epibacterial isolates from each seaweed host replicate of *F. Serratus* (green) and *P. Palmata* (red) within native environments.

To test whether bacteria isolated from *P. palmata* and *F. serratus* experienced greater fitness within native over non-native Seaweed-Derived Medium, we conducted a reciprocal transplant experiment where optical density measurements were used to quantify growth (fitness). For bacterial isolates originating from both seaweed species, higher average fitness occurred in native compared to non-native media (i.e. there was a statistically significant interaction between origin and growth environment, F_(1, 202)_ = 47.31, *P <* .001; Fig. 4A; Fig. S6). In *Palmaria* Seaweed-Derived Medium, *Palmaria*-derived isolates outperformed *Fucus*-derived isolates with a moderate effect size (mean difference = 0.45, 95% CI: 0.27 to 0.64, *P <* .0001). Meanwhile, no significant difference in fitness was observed between either isolate type within *Fucus* Seaweed-Derived Medium (mean difference = −0.016, 95% CI: −0.20 to 0.17, *P =* 0.86), indicating that *Palmaria*-derived isolates show a stronger preference for their native environments than *Fucus*-derived isolates.

Notably, there were substantial differences in the relative/delta fitness of bacterial isolates between the individual seaweed hosts. Overall, differences among host replicates contributed 11.2% to the total variation, and epibacterial fitness showed significant variation across individual host replicates for both seaweed species (*P. palmata* (Kruskal-Wallis: (H(5) = 25.37, *P =* .0001)); *F. serratus* (ANOVA): (F(5, 76) = 2.48, *P* = .039). Although the majority of isolates, 67% of *Fucus*-derived and 70% of *Palmaria*-derived, achieved higher growth on their native Seaweed-Derived Medium, across all host replicates, some isolates performed better in non-native environments. Indeed, in one instance (i.e. replicate 9, Fig. 4B), the majority of isolates derived from a single host had higher fitness in the non-native growth environment.

## Discussion

Whilst (amplicon) sequencing has provided high-resolution overviews into the distribution of taxa in natural environments and lab experiments have shed light on the ecological and evolutionary forces structuring spatial diversity, a combination of sampling and controlled lab experiments is needed to elucidate drivers of divergent selection and local adaptation. However, to estimate whether epibacteria are locally adapted to their host, it is important to culture as large a fraction of the microbiome as possible. Ideally, live seaweeds would be sterilised, followed by inoculation with native or non-native bacterial communities. Although protocols are available for seaweed sterilisation using antibiotics [50] or disinfectants [51], there is no evidence based on the published literature or our own experience (unpublished data) that the seaweed species used here can be kept in prolonged axenic culture. As an alternative, we developed host-derived media to simulate ecologically relevant host chemical environments and show that this approach recovered bacterial communities with compositions more similar to uncultured bacterial samples than those recovered using generic Marine Broth agar. These results suggest that host-specific media can provide a more realistic depiction of native bacterial communities and the approach may, therefore, be useful in a range of study systems (e.g. plants and phytoplankton) where ease of experimental manipulation and the isolation of host-associated bacterial communities is required.

For *F. serratus* and *P. palmata,* epibacterial isolates exhibited significantly higher fitness within native compared to non-native host environments and when compared to isolates transplanted from the other host species, a pattern consistent with local adaptation. Bacterial fitness, however, varied between host seaweed replicates, indicating that the extent of host-specialisation is dependent on individual hosts and/or the diversity of bacteria present. As bacterial performance was enhanced within native environments, this suggests bacteria may evolve increased fitness to their hosts and, therefore, communities are likely not structured due to chance colonisation of locally abundant strains but by host-specific selection, raising the possibility that host-bacterium coevolution may be more prevalent in marine systems than previously appreciated.

Nonetheless, as with any experimental system, our design had several shortcomings. Since we were not able to transplant bacterial communities between live seaweeds, it was not possible to replicate live holobiont interactions (between host and epibacteria, and between different epibacteria) [52] nor measure the fitness of host seaweeds. Consequently, our study stands in contrast to work focusing on host fitness, for instance, experiments replacing native gut microbiomes with non-native microbiomes derived from related host species [53]. Furthermore, while outperforming generic Marine Broth agar, Seaweed-Derived Medium achieved far from a 100% culturability rate, as autoclaving and processing steps may alter or degrade some host-specific compounds [54], and culturable isolates encompass only a subset of the true microbial diversity. Therefore, while this study provides insights into the interactions of a host-microbe system and a methodology for investigating microbial ecology under experimental conditions, these results only capture a fraction of the true potential of host selection and microbial adaptation within the seaweed holobiont.

Future work is needed to overcome methodological hurdles, which could then help in answering a host of open questions on microbial local adaptation. These questions include disentangling whether distinct microbiomes are the result of coevolution or simply the result of more distantly related hosts sharing fewer relevant traits [54], the degree to which there is functional redundancy in epibacterial phylogenetic variation [55] or whether there is significant intra-specific variation in microbiome functioning [56].

Our findings demonstrate that seaweed epibacteria exhibit local adaptation to host-supplemented agar environments, with significant structuring of bacterial communities occurring due to host-specific growth requirements. Since microbiomes are crucial to the functioning and health of seaweed hosts [1], it is possible that locally adapted microbes can bestow greater fitness to their hosts under stressful conditions, enabling hosts to specialise and endure in unfamiliar niches [6, 57]. Understanding the dynamics of microbiome assembly is therefore key for future improvements in host survivorship, which is of crucial importance to ecosystem functioning and aquaculture. While this study provides insights into the interaction of a host-microbiome system, isolates were investigated independent of their wider community and living host. More research is therefore needed to test the true potential of host selection and possible microbial adaptation within the seaweed holobiont.

## Supplementary Material

Supplementary_Materials_ycaf205

## Data Availability

All 16S rRNA gene amplicon sequencing data generated during the current study are available in the National Center for Biotechnology Information Sequence Read Archive, under BioProject PRJNA1223129 (https://www.ncbi.nlm.nih.gov/sra/PRJNA1223129). Other datasets and scripts supporting the conclusions of this article are available in the MicrobiomeLocalAdaptation repository at https://github.com/shaunacorr/LocalAdaptationinBacteria.
