# Supplementary material for: Reciprocal transplantation experiments reveal local adaptation of seaweed-associated bacteria
Source: ISME Commun. 2025 Nov 10;5(1):ycaf205. doi: 10.1093/ismeco/ycaf205 (PMC12645841; doi:10.1093/ismeco/ycaf205)
Supplement: Supplementary_Materials_ycaf205 [file supplementary_materials_ycaf205.pdf]

Reciprocal transplantation experiments reveal local adaptation of seaweed-associated bacteria – Supplementary Materials

Shauna Corr[1,2*], Chris Lowe[3] & Michiel Vos[1]

[1] European Centre for Environment and Human Health, University of Exeter Medical School, Environment and Sustainability Institute, Penryn, TR10 9FE, United Kingdom

[2] Marine Ecology and Society, Plymouth Marine Laboratory, Plymouth, PL1 3DH, United Kingdom

[3] The Cornish Seaweed Company Ltd, Rosuick Farm, St Martin, Helston, TR12 6DZ, United Kingdom

***** Corresponding Author: sc1108@exeter.ac.uk

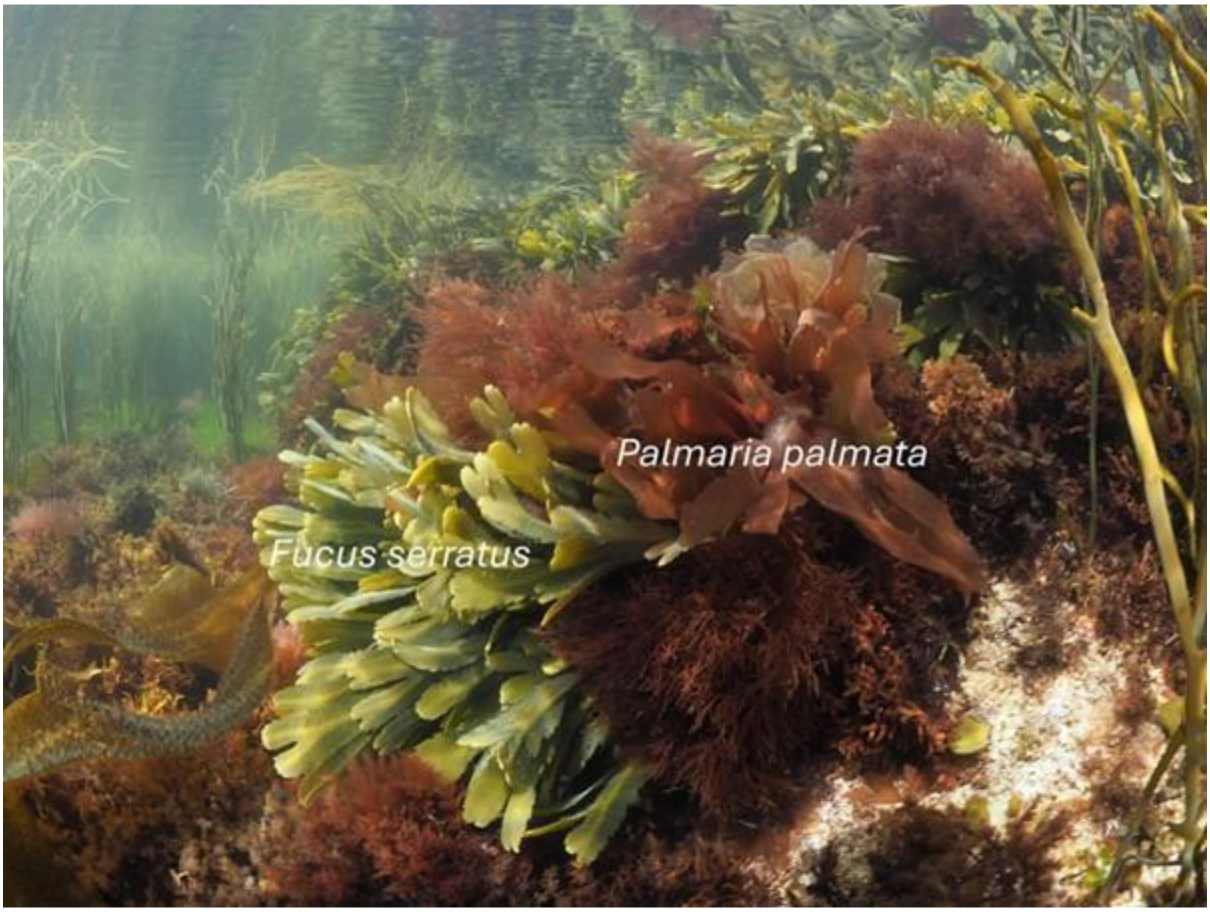

Figure S1: Photo of *F. serratus* and *P. palmata* coexisting within the same rockpool.

Table S1: Imprinting sample weights (1g ± 0.05g) and surface areas (cm^2) for culturing comparison between Seaweed Derived Medium and Marine Broth Agar for each seaweed replicate.

Seaweed Replicate	Seaweed Derived Medium		Marine Broth Agar	
	Weight (1g ± 0.05g)	Surface Area (cm^2)	Weight (1g ± 0.05g)	Surface Area (cm^2)
Fucus 1	0.95	15.81	1.01	15.50
Fucus 2	1.05	10.81	1.02	14.85
Fucus 3	1.00	16.09	1.04	12.85
Fucus 4	1.04	18.14	1.02	16.21
Fucus 5	1.05	11.15	1.05	18.49
Fucus 6	1.05	17.20	0.98	16.25
Palmaria 1	0.95	18.53	0.95	20.75
Palmaria 2	1.05	29.78	0.95	32.39
Palmaria 3	1.00	28.54	0.97	31.01
Palmaria 4	1.05	23.83	1.02	22.63
Palmaria 5	0.98	22.74	0.95	19.08
Palmaria 6	1.02	32.92	0.95	29.63

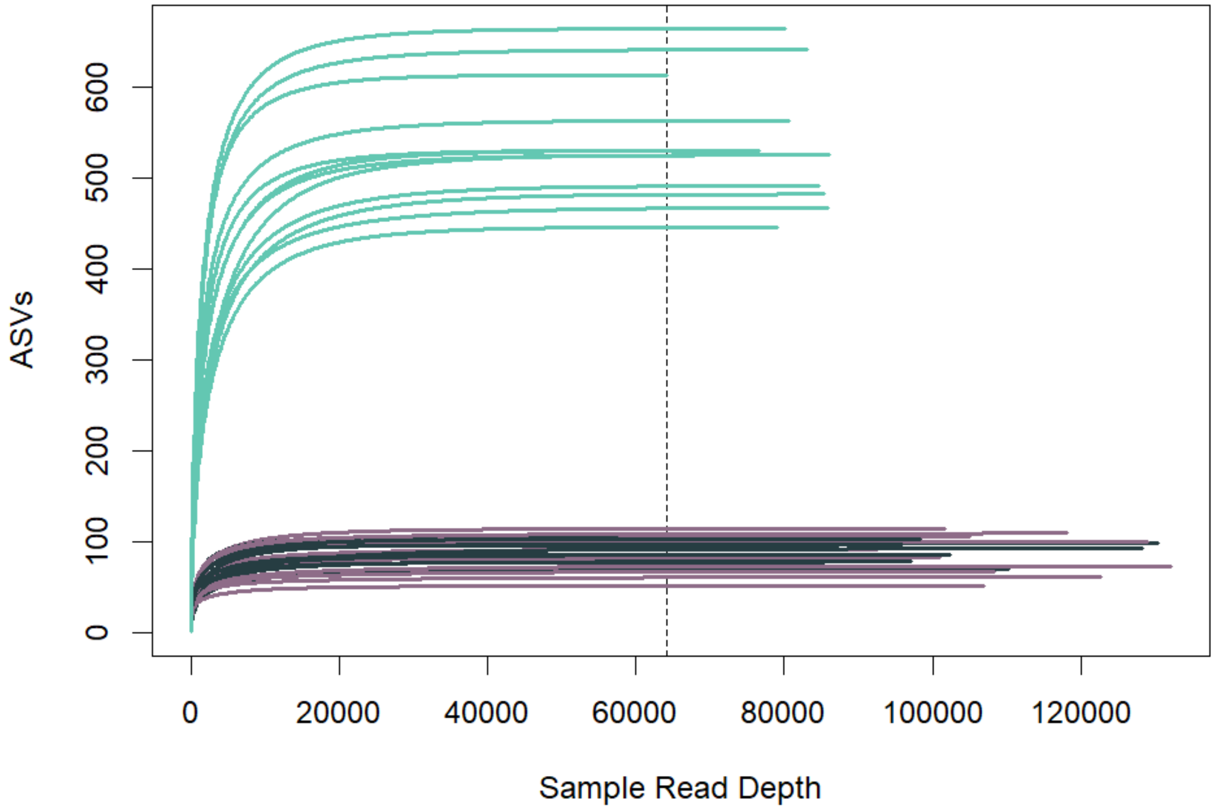

Figure S2: Rarefaction curve of sample sequencing depth from the comparison of uncultured bacterial community samples (blue), bacterial community samples obtained via Seaweed-Derived Medium (purple), and via Marine Broth Agar (black). Each curve corresponds to a (replicate) sample. Plateaus suggest sufficient sequencing to capture the majority of the diversity present. Minimum sampling depth is marked at 64,124.

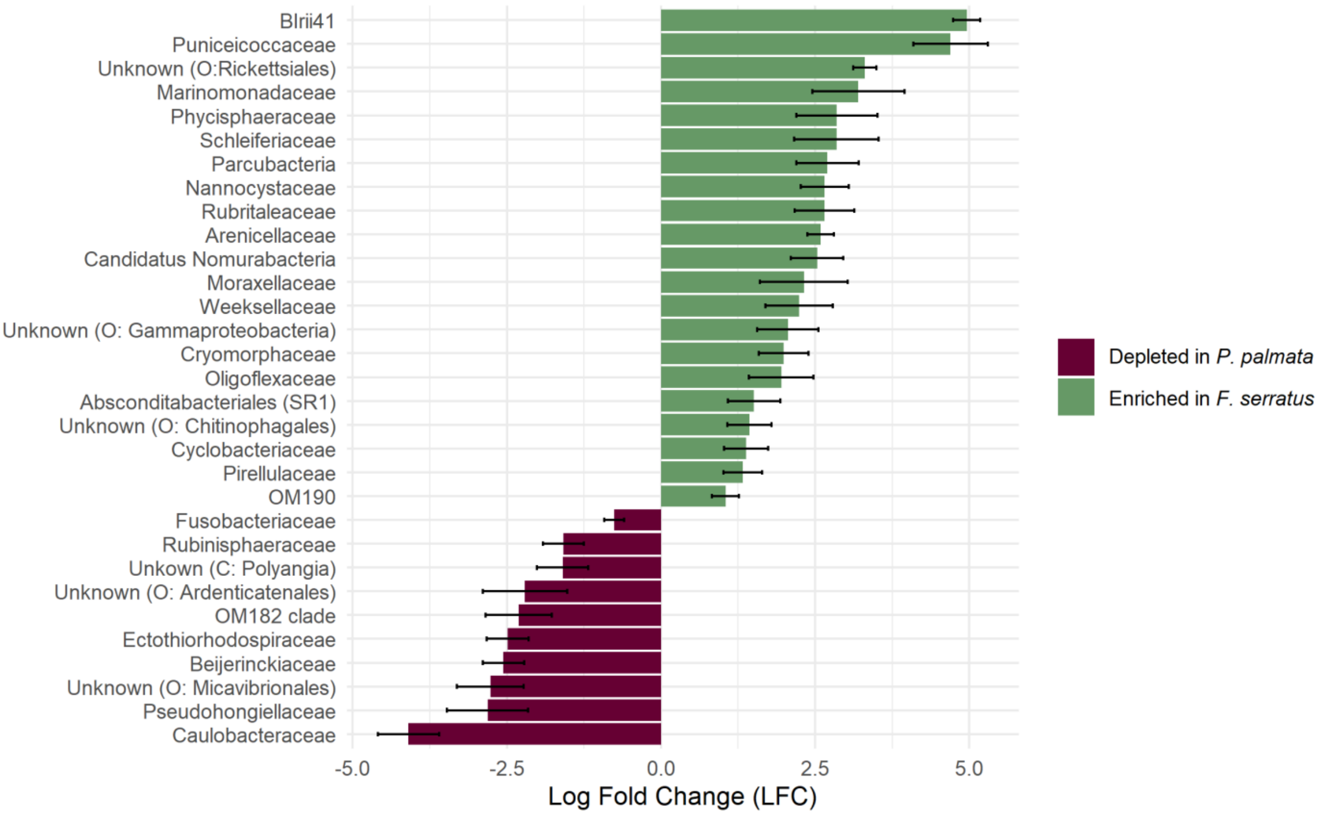

Figure S3: ANCOMBC analysis (Mandal *et al*., 2015; Lin and Peddada, 2020) of epibacterial families with differential abundance ($p < 0.05$) in uncultured *F. serratus* bacterial community versus uncultured *P. palmata* bacterial community samples.

Table S2: Pairwise comparisons of alpha and beta diversity metrics of Seaweed-Derived Medium (SDM), Marine Broth Agar (MB) and uncultured bacterial community (UN) treatments. Shannon diversity was compared using Wilcoxon rank sum exact tests at all taxonomic levels. Pairwise PERMANOVAs of Weighted UniFrac distances were used to compare beta diversity at genus level (*P* values adjusted using the *fdr* method (Benjamini and Hochberg, 1995)).

**Species**	**Treatment**	**Phylum**		**Class**		**Order**		**Family**		**Genus**		**Weighted UniFrac: Pairwise PERMANOVA *p-value***
		**Shannon Diversity Mean ± SD**	***Wilcox Rank Sum p-value***	**Shannon Diversity Mean ± SD**	***Wilcox Rank Sum p-value***	**Shannon Diversity Mean ± SD**	***Wilcox Rank Sum p-value***	**Shannon Diversity Mean ± SD**	***Wilcox Rank Sum p-value***	**Shannon Diversity Mean ± SD**	***Wilcox Rank Sum p-value***	
F. serratus	SDM	0.63 ± 0.10	**<0.01**	0.80 ± 0.12	**<0.01**	1.12 ± 0.16	**<0.01**	1.33 ± 0.24	**<0.01**	2.46 ± 0.39	**<0.01**	**<0.01**
	UN	1.37 ± 0.14		1.63 ± 0.17		2.57 ± 0.27		2.84 ± 0.33		4.88 ± 0.58		
	SDM	0.63 ± 0.10	**<0.01**	0.80 ± 0.12	**<0.01**	1.12 ± 0.16	**<0.01**	1.33 ± 0.24	0.559	2.46 ± 0.39	0.874	**<0.01**
	MB	0.15 ± 0.10		0.23 ± 0.11		0.42 ± 0.21		1.44 ± 0.26		2.36 ± 0.35		
	MB	0.15 ± 0.10	**<0.01**	0.23 ± 0.11	**<0.01**	0.42 ± 0.21	**<0.01**	1.44 ± 0.26	**<0.01**	2.36 ± 0.35	**<0.01**	**<0.01**
	UN	1.37 ± 0.14		1.63 ± 0.17		2.57 ± 0.27		2.84 ± 0.33		4.88 ± 0.58		
	Fucus UN	1.37 ± 0.14	0.332	1.63 ± 0.17	0.422	2.57 ± 0.27	0.422	2.84 ± 0.33	0.749	4.88 ± 0.58	1	**<0.01**
	Dulse UN	1.25 ± 0.19		1.56 ± 0.18		2.39 ± 0.16		2.65 ± 0.14		4.82 ± 0.52		
P. palmata	SDM	0.59 ± 0.16	**<0.01**	0.73 ± 0.22	**<0.01**	0.89 ± 0.20	**<0.01**	1.19 ± 0.14	**<0.01**	2.39 ± 0.25	**<0.01**	**<0.01**
	UN	1.25 ± 0.19		1.56 ± 0.18		2.39 ± 0.16		2.65 ± 0.14		4.82 ± 0.52		
	SDM	0.59 ± 0.16	**<0.05**	0.73 ± 0.22	**<0.05**	0.89 ± 0.20	**<0.01**	1.19 ± 0.14	0.937	2.39 ± 0.25	0.0685	**<0.05**
	MB	0.30 ± 0.16		0.33 ± 0.17		0.41 ± 0.17		1.23 ± 0.27		2.00 ± 0.29		
	MB	0.30 ± 0.16	**<0.01**	0.33 ± 0.17	**<0.01**	0.41 ± 0.17	**<0.01**	1.23 ± 0.27	**<0.01**	2.00 ± 0.29	**<0.01**	**<0.01**
	UN	1.25 ± 0.19		1.56 ± 0.18		2.39 ± 0.16		2.65 ± 0.14		4.82 ± 0.52		

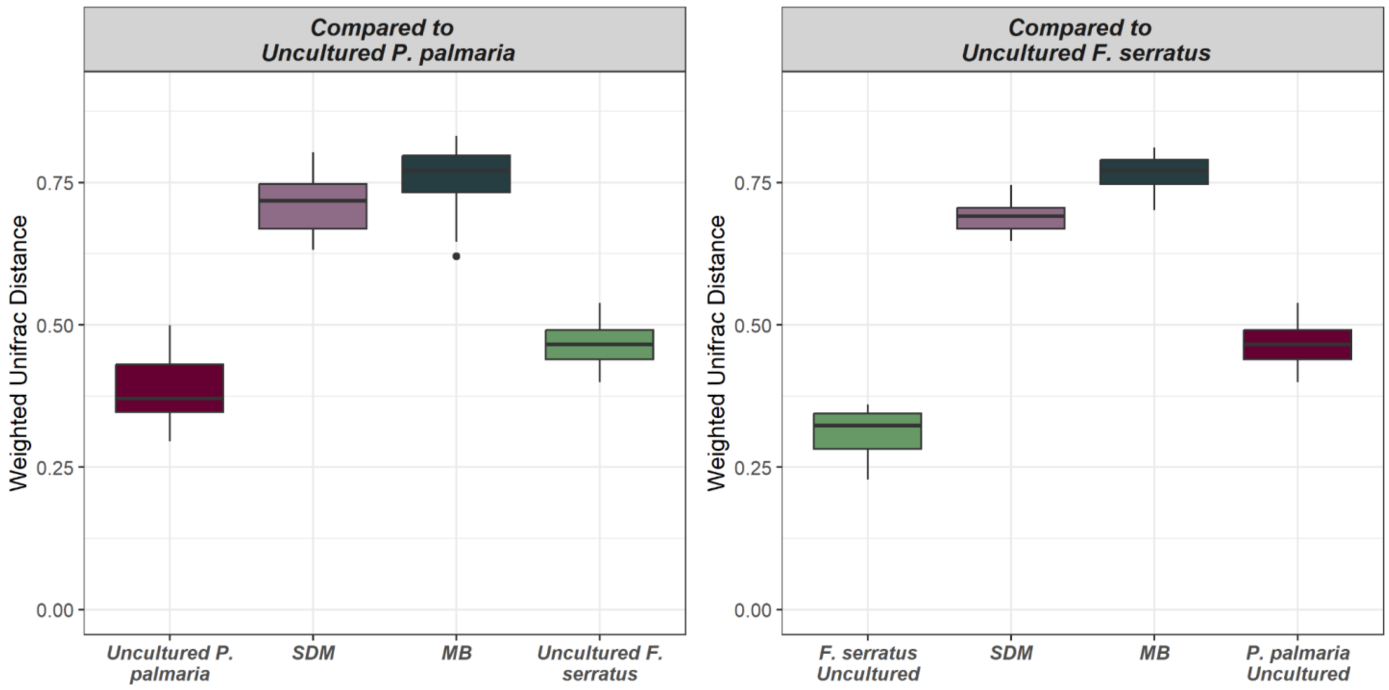

Figure S4: Pairwise PERMANOVA comparison plot of weighted UniFrac distances of uncultured *P. palmata* (left) and *F. serratus* (right) samples, with uncultured bacterial community (*P. palmaria*: red, *F. serratus*: green), Seaweed-Derived Medium (SDM: purple), and Marine Broth samples (MB: black).

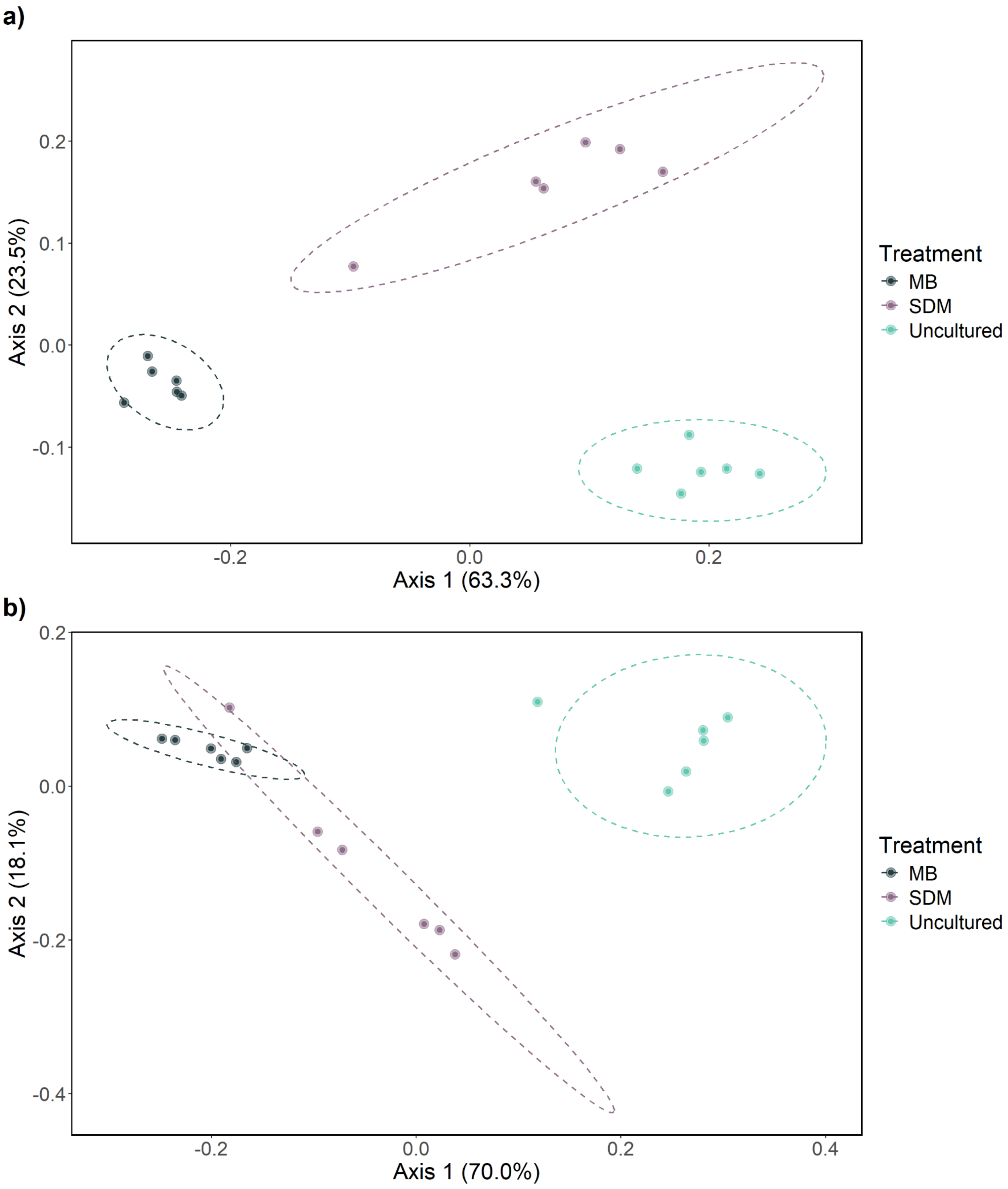

Figure S5: Principal Coordinate Analysis plot based on weighted UniFrac distances of uncultured (blue) and cultured bacterial communities via Seaweed-Derived Medium (SDM: purple) and Marine Broth Agar (MB: black) of (a) *Fucus serratus* and (b) *Palmaria palmata* samples. 95% confidence levels are displayed with ellipses.

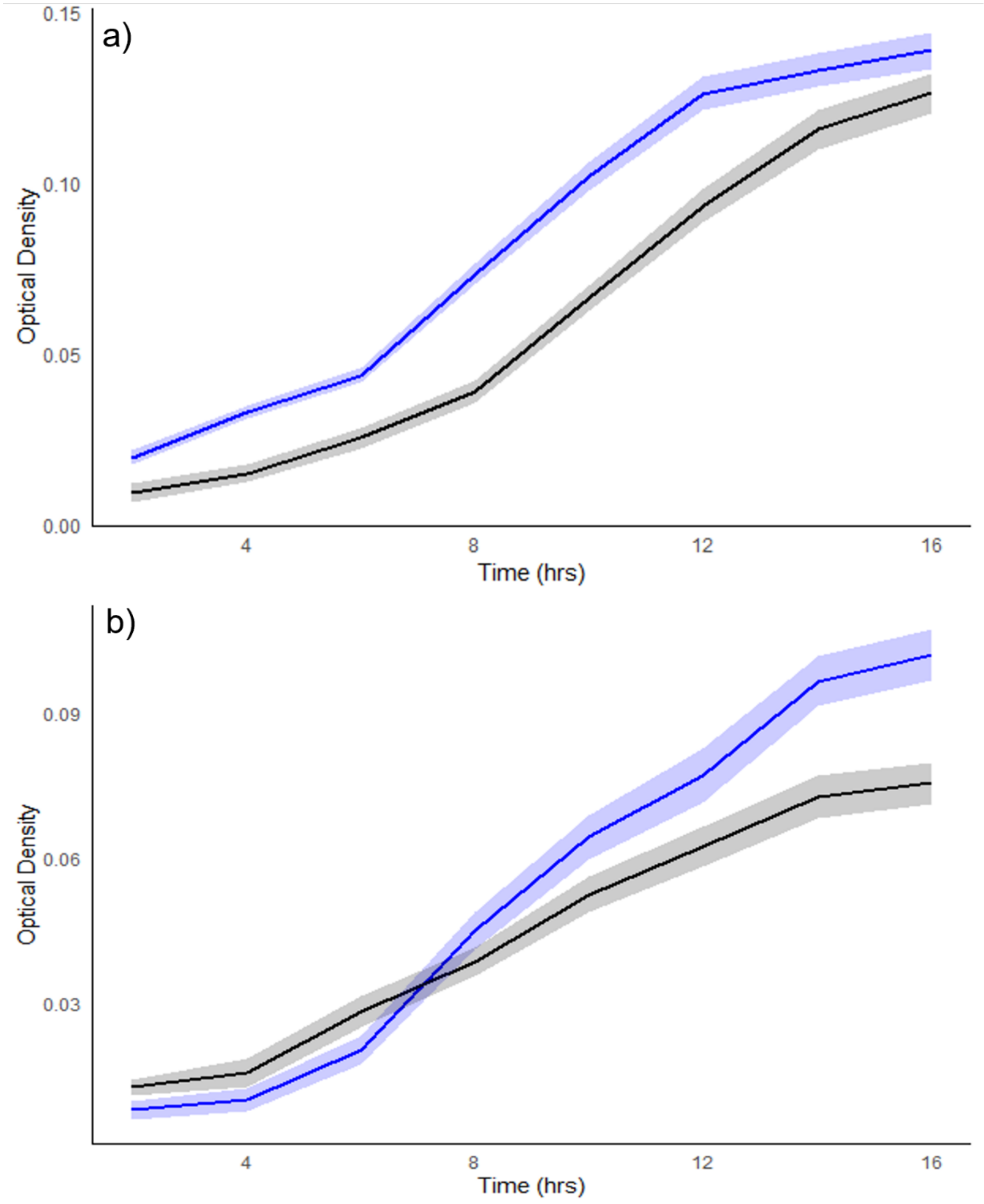

Figure S6: Average (mean ± SE) growth curves of *P. palmata* (a) and *F. serratus* (b) epibacterial isolates in either a native (blue) or non-native (black) environment.

References

Lin H, Peddada SD. Analysis of compositions of microbiomes with bias correction. *Nat Commun* 2020;11:3514. 10.1038/s41467-020-17041-7

Mandal S, Van Treuren W, White RA, Eggesbø M, Knight R, Peddada SD. Analysis of composition of microbiomes: a novel method for studying microbial composition. *Microb Ecol Health Dis* 2015;29:27664. 10.3402/mehd.v26.27663
